# Oscillatory interlayer coupling in spin Hall systems

**DOI:** 10.1038/s41598-018-20685-7

**Published:** 2018-02-02

**Authors:** A. M. Gonçalves, F. Garcia, H. K. Lee, A. Smith, P. R. Soledade, C. A. C. Passos, M. Costa, N. M. Souza-Neto, I. N. Krivorotov, L. C. Sampaio, I. Barsukov

**Affiliations:** 1 Centro Brasileiro de Pesquisas Físicas, Rio de Janeiro, Brazil; 2 University of California, Irvine, CA USA; 3 Universidade Federal do Amapá, Amapá, Brazil; 4 Universidade Federal do Espírito Santo, Espírito Santo, Brazil; 5 Laboratório Nacional de Luz Síncrotron, Campinas, Brazil; 6 University of California, Riverside, CA USA

## Abstract

Many spintronics applications consist of ultrathin magnetic and nonmagnetic multilayers and require an in-depth understanding of interfacial magnetism and spin transport. Here, we study permalloy/copper/platinum multilayer systems. We find that magnetic damping, perpendicular anisotropy, and proximity magnetization exhibit correlated oscillations as a function of the copper thickness. We ascribe these observations to an oscillatory interlayer coupling between permalloy and platinum. Such interlayer coupling may have a significant impact on the performance of spintronics applications.

## Introduction

Recent advances in spintronics facilitate efficient manipulation of the spin degree of freedom^1–5^, electrical detection of static and dynamic magnetic states^6,7^, and significant improvement of energy-efficiency by energy harvesting^8,9^. Many nano-scale spintronic devices contain a heavy-metal layer employed for creating or detecting spin currents from an adjacent ferromagnetic layer^10,11^ using spin Hall effect^12,13^. These layers are often separated by a thin metallic layer with low spin scattering rate^14,15^. Understanding interfacial spin transport and magnetism^1,16^ in such systems is an essential prerequisite for spintronics applications^6,10,11^.

Spin Hall systems consist of a ferromagnetic (FM) layer and a nonmagnetic (normal metal, NM) layer with large spin Hall angle, such as platinum^17^. By supplying an electrical current through the NM, a pure spin current is injected into the FM that can be used to tune magnetic damping^6,18^. This effect is employed in spin Hall based magnetic switching^2,19^ and auto-oscillators^4,5^. On the other hand, magnetic excitations in the FM inject a spin current into the NM, that can be detected using inverse spin Hall effect^6^. Moreover, application of a temperature gradient perpendicular to the interface creates an interfacial spin current that gives rise to a spin Seebeck voltage in the NM and to a spin Seebeck torque in the FM^8,9^. FM/NM interfaces may also exhibit interfacial Dzyaloshinskii-Moriya interaction (IDMI)^1,20–22^ which can be employed for controlling chirality of magnetization structures and for stabilizing magnetic Skyrmions^23^.

Direct proximity of NM to FM bears considerable complications. The NM is typically a heavy-metal with large spin-orbit coupling and can cause a significant increase of magnetic damping^17,24^ and perpendicular magnetic anisotropy^25,26^ in the adjacent FM layer. Interfacial spin memory loss can significantly hinder spin transport between FM and NM^14,27^. Furthermore, the NM layer can acquire proximity induced magnetization, as has been shown for NM materials with high Stoner-enhanced susceptibility^28,29^. Proximity magnetization can lead to undesirable magnetoresistive effects^15^, such as anisotropic magnetoresistance and anomalous Nernst effect^30,31^. Moreover, it may reduce the spin Hall efficiency of the NM^15^.

A common approach to avoid these complications is the insertion of a thin intermediary layer (IL) between FM and NM^27,32^. This intermediary layer consists of a metal with low spin-orbit coupling^24,33^, such as copper, ensuring a low spin-scattering rate to facilitate spin transport across the layers^14,17,34^. However, recent works have presented evidence for a significant departure from this picture. For instance, Montoya *et al*.^35^ have shown the effect of enhanced Gilbert damping in Fe/Au/Pd systems due to formation of quantum well states in the Au intermediary layer. Bailey *et al*.^28^ have studied [Permalloy/Cu/Pd/Cu]_20_ multilayers and observed proximity magnetization in Pd, suggesting the presence of indirect exchange between Permalloy (Py) and Pd layers. Okabayashi *et al*.^36^ have shown that perpendicular magnetic anisotropy can be induced in a Co layer by a Pt layer through <0.7 nm intermediary Cu layer. These findings show that spin transport through an intermediary layer may involve more complex mechanisms and requires a closer attention^17,37^.

Šimánek and Heinrich^38^ have proposed a theoretical model based on time retarded response of spin dependent scattering, that points towards an indirect exchange coupling through the intermediary layer. Barati and Cinal^39^ have carried out a fully quantum-mechanical calculation to show nonlocal enhancement of the Gilbert damping in the FM layer by the spin-orbit coupling in the NM, which presents small oscillations with the thickness of the intermediary layer. However at present, a consistent theoretical model making a connection between an indirect exchange coupling and the effects on the damping, magnetic anisotropy, and proximity magnetization is not available. Moreover, these phenomena have not yet been observed in a single set of samples, leaving their mutual relation open for speculations.

Here, we investigate spin Hall systems consisting of Py/Cu/Pt multilayers with variable Cu layer thickness. In this experimental study, we analyze magnetic anisotropy, damping, and proximity magnetization and find their correlated oscillatory dependence on the Cu layer thickness. We present a heuristic model to explain the observed phenomena.

## Results

One set (set 1) of samples was grown by sputter deposition and presents multilayers consisting of Si/SiOx(substrate)/Py(10)/Cu(*t*)/Pt(3), with thicknesses given in nanometers. Within this set of samples, the thickness *t* of the Cu layer varies in a range of 0–3 nm. In addition, another set (set 2) of samples with the structure (0001)Al_2_O_3_(substrate)/Pt(4.5)/Cu(*t*)/Py(5)/Al(3.5) was grown in a different deposition chamber. We stress that the same growth procedure was repeated for each sample within one sample set.

**Figure 1:**
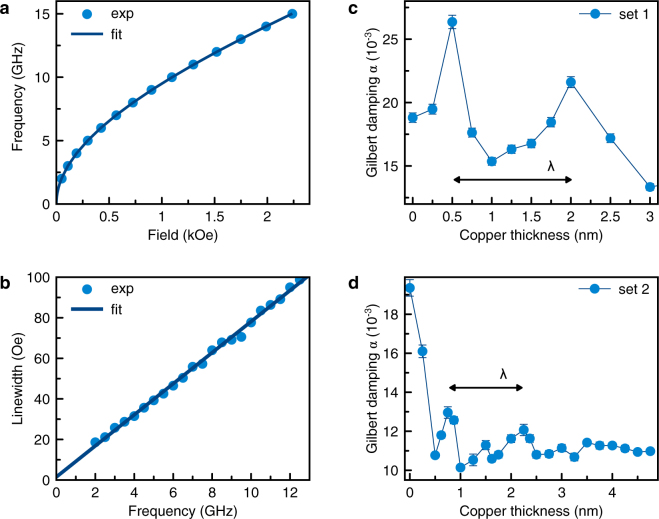
(**a**) Typical ferromagnetic resonance data: frequency–resonance field relation. (**b**) Linewidth as a function of frequency. (**c**) Gilbert damping *α* as a function of Cu thickness *t* for the set 1 of samples grown on oxidized silicon (Si/SiOx). (**d**) Gilbert damping for the set 2 of samples grown on sapphire (Al_2_O_3_).

Each sample was characterized by broadband ferromagnetic resonance (FMR)^40,41^ (Supplementary Figs. 1 and 2). The FMR frequency *f* as a function of the in-plane field is shown in Fig. 1a, it follows the expected thin-film behavior (Supplementary Fig. 3)^42^ described by the Kittel equation:

1 $$f=\frac{|\gamma |}{2\pi }\sqrt{{H}_{{\rm{r}}}({H}_{{\rm{r}}}+{H}_{{\rm{eff}}})},$$

where the gyromagnetic ratio is |*γ*| = 2*π* · 2.911 GHz/kOe and *H*_eff_ is the effective perpendicular anisotropy field, arising from the magnetic shape anisotropy and contributions of the perpendicular magnetic anisotropy (PMA). *H*_r_ is the resonance field.

In Fig. 1b, the FMR linewidth is shown. It presents a linear trend as a function of frequency (Supplementary Fig. 4). The y-axis intercept Δ*H*_0_ is negligibly small. Such behavior indicates negligibly small magnetic inhomogeneity and two-magnon scattering^43,44^. In the absence of field dragging^43^, the Gilbert damping parameter *α* can be extracted^43,45^ according to:

2 $${\rm{\Delta }}H={\rm{\Delta }}{H}_{0}+\frac{2\pi f}{|\gamma |}\alpha $$

The Gilbert damping is usually discussed to consolidate two contributions: (i) due to intrinsic damping and (ii) due to the transfer of angular momentum from Py into Pt layer through the Cu layer by spin pumping^14,17,46^. We consider the intrinsic damping, being a material parameter, as constant for all samples within a set of samples because of the fixed growth conditions. The spin pumping contribution is expected to be a monotonically decreasing function of the Cu thickness^17,27^. However, we observe a strongly non-monotonic behavior of the Gilbert damping. As shown in Fig. 1c, two strongly pronounced peaks of *α* appear at *t* = 0.5 nm and *t* = 2 nm. Moreover, the data suggests a much smaller peak at *t* = 1.25 nm.

This result urges for reexamining the above-mentioned picture of interfacial spin transport in multilayer systems. The peaks in Fig. 1c appear to be equidistant, which suggests an oscillatory behavior of the observed damping enhancement. We may assume a coupling of spins at the Py/Cu and Cu/Pt interfaces^28,35,37^ through the Cu layer. Such coupling would be an analog of the RKKY interlayer exchange coupling between two ferromagnets separated by a nonmagnetic buffer^37,47^, such as e.g. in [Co/Cu]_*n*_ multilayers studied by Parkin *et al*.^37^. The RKKY interaction presents an oscillatory behavior, leading to alternating parallel and anti-parallel coupling magnitude with increasing thickness of the intermediary layer (Cu). Electrons in Cu with the wavevectors perpendicular to the interface, which connect the points on the Fermi surface with opposite velocities (critical wavevectors)^47^, facilitate the coupling through the Cu layer^28,35,38,46,47^.

Depending on the crystallographic direction of Cu, there can be several critical wavevectors^47^. The layers in sample set 1 are grown on oxidized silicon and present a weak (111) crystallographic texture^48–50^. Several critical wavevectors therefore contribute to the exchange coupling and obscure a sharp periodic behavior^37,47,51^. However, the sample set 2 is grown on single crystal sapphire substrates and therefore presents a strong (111) crystallographic texture^49,50,52,53^. This orientation has been chosen because for the 〈111〉 directions in Cu, only one single critical wavevector exists^47^. The Gilbert damping of sapphire-based samples is shown as a function of Cu thickness in Fig. 1d. It presents a clear oscillatory behavior with several well pronounced peaks. The peaks are found to be equidistant. The separation between two neighboring peaks amounts to approximately 0.7 nm. This value is very close to the half-period *λ*/2 = 0.625 nm of the RKKY interaction for (111)Cu reported by Mosca *et al*.^51^; similar values have been reported in other studies^47^. The half-period of the RKKY interaction corresponds to the change in Cu thickness between a parallel (P) and a neighboring anti-parallel (AP) coupling regime. Our data therefore suggest that the damping increases for both parallel and anti-parallel coupling.

**Figure 2:**
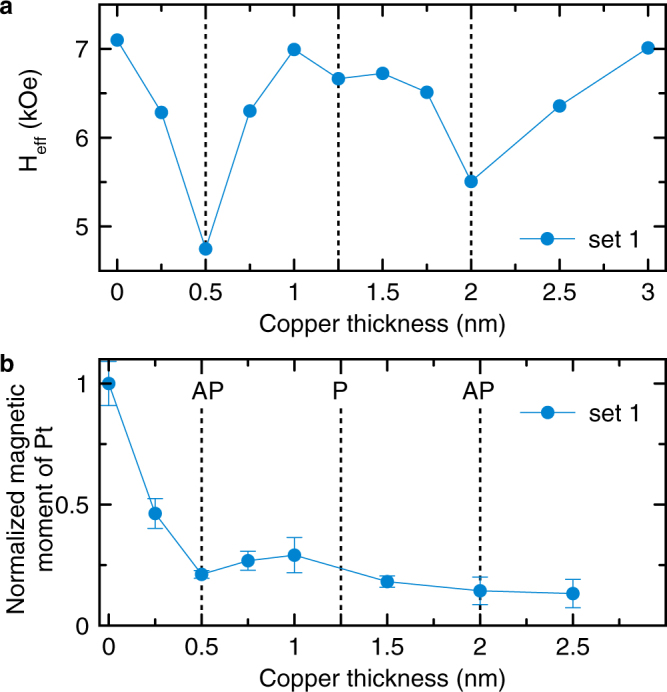
(**a**) Effective perpendicular anisotropy field as a function of Cu thickness. (**b**) Normalized magnetic moment induced in the Pt layer. The vertical lines indicate critical Cu thickness of parallel (P) and anti-parallel (AP) coupling regimes.

The RKKY-type interlayer exchange model^47^ is well established for systems consisting of FM/IL/FM layers. In our samples one FM layer is substituted by platinum^54^, and the model is not directly applicable. Remaining within the heuristic model for the interlayer coupling between the Py/Cu and Cu/Pt interfacial spins, we assume that the strong spin-orbit coupling of Pt is mediated to and has an effect on Py. In particular, the spin-orbit coupling should give rise to a perpendicular magnetic anisotropy (PMA) in Py^1,25,36,55–57^. Figure 2a shows the effective perpendicular anisotropy field *H*_eff_ as a function of Cu thickness for the sample set 1. Again, non-monotonic behavior is observed. Two well pronounced dips are at *t* = 0.5 nm and *t* = 2 nm. A smaller dip is apparent at *t* = 1.25 nm. These dips correspond to increased perpendicular magnetic anisotropy and appear at the same positions as the peaks in Gilbert damping shown in Fig. 1c.

The peaks of the Gilbert damping and PMA appear in a correlated oscillatory manner with a separation equal to the half-period of the RKKY-type interaction. This observation suggests that *α* and PMA increase for both parallel and anti-parallel coupling. However, there is an apparent asymmetry between these two coupling regimes. It is best demonstrated by the Gilbert damping data shown in Fig. 1c. On the one hand, the peak amplitudes decrease with increasing Cu thickness, as expected for the interlayer coupling^37,47,58^. On the other hand, the peak amplitudes alternate, starting with a larger amplitude at *t* = 0.8 nm. This observation raises the question, which coupling regime, P or AP, has a bigger impact on *α* and PMA. We address this question by assessing the induced magnetic moment of Pt.

X-ray resonant magnetic scattering (XRMS)^59^ was carried out at Brazilian Synchrotron Light Laboratory (LNLS) at the dispersive beam line DXAS. The measurements were performed in a reflectivity mode at L3 edge of Pt at room temperature, keeping fixed the X-ray circular polarization (~80%) and switching the magnetic field between 1 T and −1 T^60^. We obtain measurements of the total induced magnetic moment *μ*_Pt_ in the Pt layer (Supplementary Note 1).

Due to the high Stoner susceptibility of Pt^29,30,61^, a magnetic moment is induced in Pt by exchange interaction that aligns Pt moments parallel to the moments of Py in direct proximity. However, even with addition of the intermediary Cu layer, magnetic moment in the NM layer was observed to persist^24,28^. In Ref.^28^, direct exchange through pinholes in the intermediary layer and dipolar (Néel) coupling were discussed and found to be unlikely the mechanisms for the induced magnetic moment in NM. In our samples, we cannot exclude the presence of pinholes in the intermediary layer^24^ and simply allow for an additional positive exchange between FM and NM. At any rate, the induced magnetic moment in NM is expected to decrease with increasing Cu thickness^24,28^.

Since the induced magnetic moment of Pt is small (~10^−1^ *μ*_B_ per atom^62^ at the interface), the measurements of *μ*_Pt_ are typically carried out on repeated multilayer systems in order to increase the signal^28^. For the present study, however, it is essential to correlate the effects of Gilbert damping, PMA, and proximity magnetic moment on the same set of samples. The XRMS measurements on the single repetition sample set 1, as shown in Fig. 2b, cannot be used to determine the absolute value of the induced magnetic moment of the layer or the magnetic moment per Pt atom, but provide sufficient relative accuracy to qualitatively assess the behavior of *μ*_Pt_ as a function of Cu thickness. The Pt moment shows a general decreasing trend, but also presents a non-monotonicity clearly visible in the *t* = 0.5–1.5 nm region. Although the data density is insufficient to declare an oscillatory behavior, the observed non-monotonicity supports the picture of alternating interlayer coupling. The magnetic moment falls rapidly at *t* = 0.5 nm and intermediately recovers at approximately *t* = 1.25 nm. This behavior suggests that in this Cu thickness region, the interlayer coupling operates in the anti-parallel regime and thus partially compensates the direct exchange. The first larger peak of Gilbert damping (Fig. 1c) and PMA at *t* = 0.5 nm (Fig. 2a) therefore corresponds to the anti-parallel coupling regime, whereas the second smaller peak at *t* = 1.25 nm corresponds to the parallel coupling regime. This picture is consistent with previous studies of magnetoresistance in [Co/Cu]_*n*_ multilayers by Mosca *et al*.^51^, where alternating coupling regimes (starting with AP) have been observed at Cu thicknesses very similar to those presented in Fig. 1d.

## Discussion

The findings in our study allow for the conclusion that the spins at the Py/Cu and Cu/Pt interfaces experience an indirect interlayer coupling through the Cu layer. The magnitude of the coupling decreases with increasing Cu thickness and, in addition, presents an oscillatory alternating behavior. The damping and the perpendicular magnetic anisotropy exhibit peaks at the critical Cu thicknesses corresponding to the extrema of the coupling magnitude (both parallel and anti-parallel). The enhanced PMA suggests that the Py interface band structure is affected by the spin-orbit interaction in Pt^1,25,36,55,56^ mediated via interlayer coupling. The oscillatory enhancement of the Gilbert damping can be caused by such modification of the Py band structure as well and/or by an enhanced spin pumping augmented by the interlayer coupling. Direct measurements^34^ of the spin pumping by inverse spin Hall effect may help to separate these two effects.

The interlayer coupling between FM and NM through an intermediary layer is observed in two very different sets of samples. The enhancement of the Gilbert damping appears at different Cu thicknesses for sample set 1 and 2. The rate of the overall decrease of the Gilbert damping with increasing Cu thickness is different. These observations are likely owed to dissimilar interfacial quality and interdiffusion in the sample sets. The oscillation periods, however, are similar, underlining the importance of the critical wavevectors in Cu. We also find larger oscillations of the Gilbert damping and PMA in the sample set 1 (Supplementary Fig. 5). A more detailed study with larger number of sample sets, accompanied by in-depth structural analysis, would be necessary to assess the observed differences. We hope that our results stimulate the development of a theoretical model to evaluate the heuristic picture and to describe the effects presented here. In particular, the question remains unanswered, why the anti-parallel coupling regime enhances the Gilbert damping and the PMA more strongly than the parallel coupling regime. A possible complication to the development of the model is the recently observed perpendicular magnetic anisotropy in copper intermediary layers, discussed in Ref.^36^. Further, it would be advantageous to investigate whether the damping and anisotropy enhancements can be decoupled, as it would promote the interlayer coupling as a tool for designing FM/IL/NM spintronic applications^32,33^. The indirect interlayer exchange coupling should also be addressed^20,63^ in the context of the interfacial Dzyaloshinskii-Moriya (IDMI) interaction^21,22^ that can be controlled by means of the intermediary layer. The interlayer coupling needs to be taken into account in studies of interfacial spin transport and may significantly impact the performance of multilayer based spintronics applications.

### Data availability

All data supporting the findings of this study are available within the article or are available from the corresponding author on reasonable request.

## Electronic supplementary material

Supplementary Information
