# Supplementary material for: Oscillatory interlayer coupling in spin Hall systems
Source: Sci Rep. 2018 Feb 2;8:2318. doi: 10.1038/s41598-018-20685-7 (PMC5797137; doi:10.1038/s41598-018-20685-7)
Supplement: Supplementary file 1 — Supplementary Information [file 41598_2018_20685_MOESM1_ESM.pdf]

Supplementary information
Oscillatory interlayer coupling in spin Hall systems

A. M. Gonçalves,[1] F. Garcia,[1] H. K. Lee,[2] A. Smith,[2] P. R. Soledade,[3] C. A. C. Passos,[4] M. Costa,[5] N. M. Souza-Neto,[5] I. N. Krivorotov,[2] L. C. Sampaio,[1] and I. Barsukov[6]

[1]*Centro Brasileiro de Pesquisas Físicas, Rio de Janeiro, Brazil*
[2]*University of California, Irvine, CA, USA*
[3]*Universidade Federal do Amapá, Amapá, Brazil*
[4]*Universidade Federal do Espírito Santo, Espírito Santo, Brazil*
[5]*Laboratório Nacional de Luz Síncrotron, Campinas, Brazil*
[6]*University of California, Riverside, CA, USA*

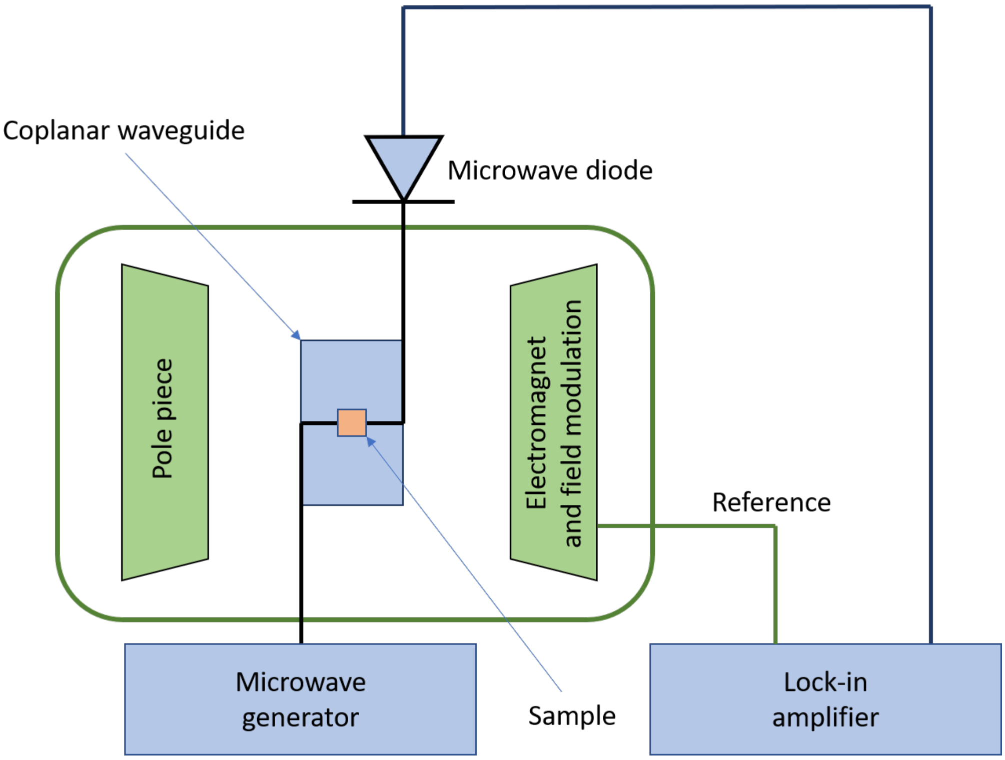

Supplementary Figure 1: Schematic of the experimental setup. Broadband ferromagnetic resonance with field modulation.

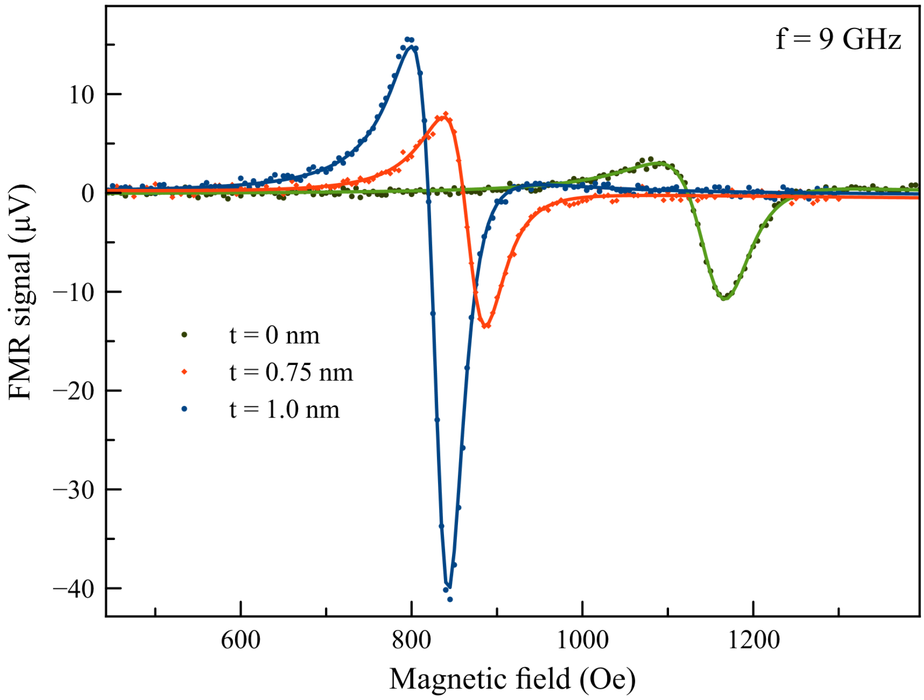

Supplementary Figure 2: Exemplary FMR spectra from sample set 2 at 9 GHz for different Cu thicknesses t with fits to the field-derivative sum of symmetric and antisymmetric Lorentzian profiles.

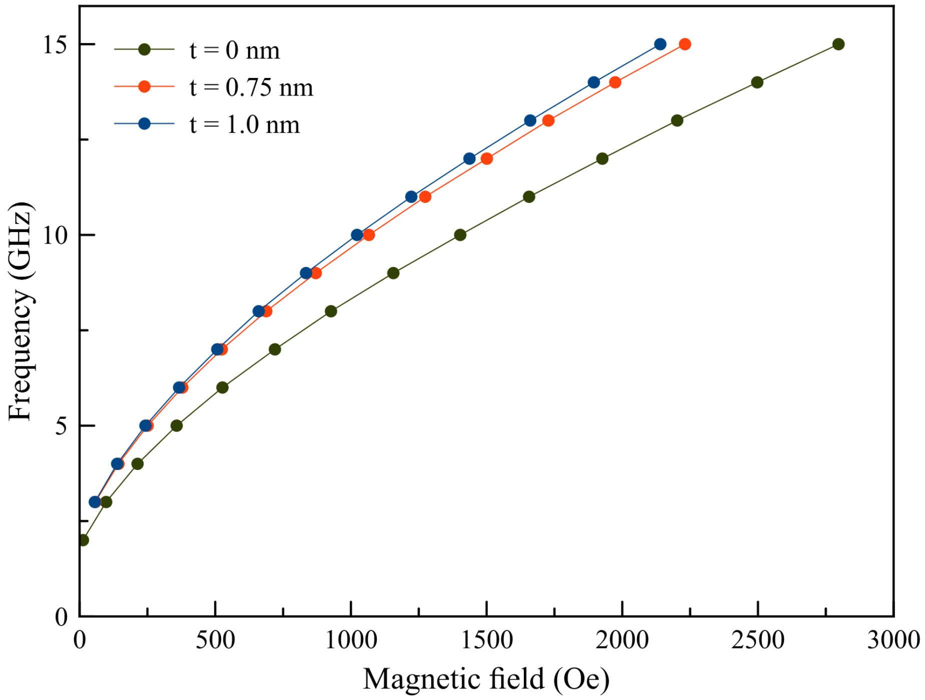

Supplementary Figure 3: Exemplary curves of resonance frequency versus field from sample set 2 for different Cu thicknesses t.

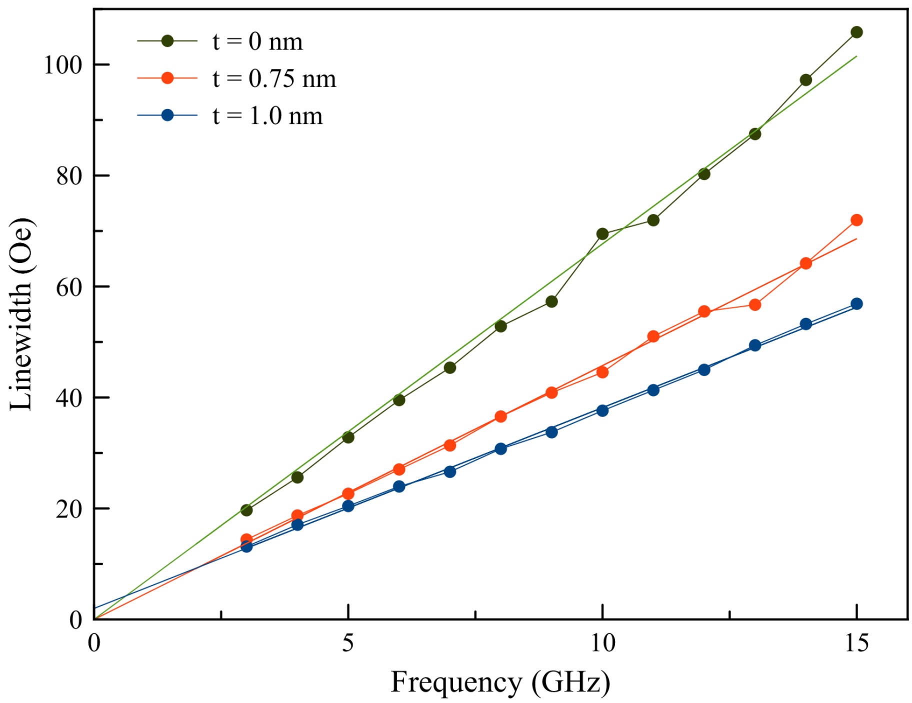

Supplementary Figure 4: Exemplary curves of linewidth versus frequency from sample set 2 for different Cu thicknesses t.

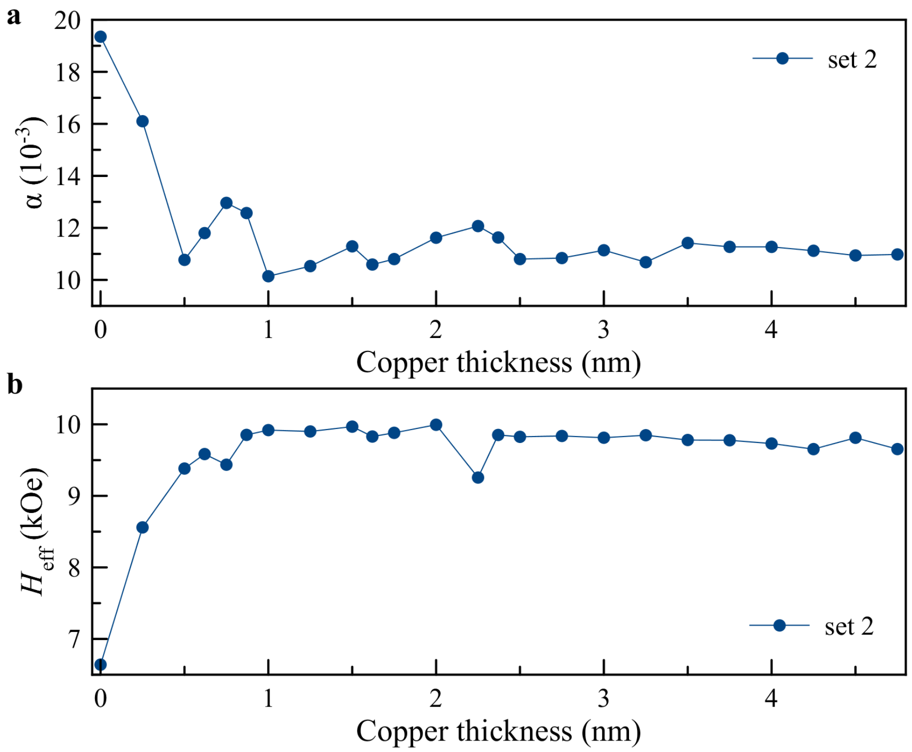

Supplementary Figure 5: (a) Gilbert damping as a function of the Cu thickness for sample set 2. (b) Corresponding effective perpendicular anisotropy field.

SUPPLEMENTARY NOTE 1

The magnetic moment of the interfacial Pt was estimated by maximum amplitude of the XRMS signal. A pair of XAS spectra was obtained in grazing incidence, the first being under a positive applied field parallel to the direction of propagation of the x-rays beam, and the second in the opposite direction. The XRMS was calculated by taking the difference of both spectra. It was recorded for at least 500 pairs of spectra for each measured sample. The final XRMS signal was obtained by averaging over all spectra pairs. The amplitude of the XRMS was calculated by fitting a Voigt function to the XRMS signal and the error bar considered was the standard deviation of the fit.
